# Efficacy of *Erwinia amylovora* and *Xanthomonas campestris* pv *campestris* phages to control fire blight and black rot *in vivo*

**DOI:** 10.1128/spectrum.00280-25

**Published:** 2025-05-16

**Authors:** Gloria Vique, Elena Mendoza-Barberá, Maria Dolores Ramos-Barbero, Pedro Blanco-Picazo, Laura Sala-Comorera, Pablo Quirós, Sergio Atares, Ignasi Salaet, Maite Muniesa, Lorena Rodríguez-Rubio

**Affiliations:** 1 Departament de Genètica, Microbiologia i Estadística, Universidad de Barcelona 200148 , Barcelona, Spain; 2 Departament de Biologia, Sanitat i Medi Ambient, Facultat de Farmàcia i Ciències de l’Alimentació, Universidad de Barcelona 368911 , Barcelona, Spain; 3 Institut d’Investigació en Nutrició i Seguretat Alimentària (INSA), Universitat de Barcelona 16724, Barcelona, Spain; 4 Departamento de I+D+i de Fertinagro Biotech S.L., Polígono Industrial La Paz 686981 , Teruel, Spain; Universidad Nacional Autonoma de Mexico - Campus Morelos, Cuernavaca, Mexico

**Keywords:** bacteriophages, lysis, cocktail, *Erwinia*, *Xanthomonas*, eco-friendly

## Abstract

Phytopathogens, such as *Erwinia amylovora* and *Xanthomonas campestris*, pose significant threats to agriculture, leading to substantial economic losses. Traditional chemical pesticides can harm soil fertility, contaminate water, and impact non-target organisms such as natural predators and pollinators, highlighting the need for sustainable pest control methods. This study explores the use of bacteriophages as biocontrol agents against *E. amylovora*, which causes fire blight, and *X. campestris* pv. *campestris*, responsible for black rot in cruciferous vegetables. Bacteriophages were isolated from urban wastewater and tested for their lytic activity against these pathogens. Three virulent phages were identified: ɸEF1 and ɸEF2 against *E. amylovora* and ɸXF1 against *X. campestris* pv. *campestris*. Genetic analysis confirmed the absence of known lysogeny-related genes, indicating that these phages are ideal candidates for biocontrol applications. *In vitro* assays demonstrated significant bacterial population reductions. Specifically, ɸEF1 killed 92.1% of the *E. amylovora* population at a multiplicity of infection (MOI) of 1 after 3 h, while ɸEF2 reduced the population by 98.1%. When combined in a 1:1 ratio, the two phages reduced *E. amylovora* populations by 99.7%, and no regrowth of resistant cells was observed, which was not the case when the phages were applied individually. ɸXF1 killed 99.9% of *X. campestris* pv. *campestris* populations at an MOI of 1 after 5 h. *In vivo* experiments using pears and kohlrabi as infection models further validated the phage effectiveness. Treated pears showed reduced fire blight symptoms, and kohlrabi plants exhibited markedly less necrosis from black rot compared to untreated controls.

**IMPORTANCE:**

Three new virulent phages have been isolated: two targeting *Erwinia amylovora* and one targeting *Xanthomonas campestris* pv. *campestris*. All phages were able to rapidly reduce the population of their corresponding phytopathogens and alleviate disease symptoms in *in vivo* plant models. These findings highlight the potential of these phages as biocontrol agents for managing bacterial plant diseases, offering an alternative to traditional chemical treatments.

## INTRODUCTION

Phytopathogens are a major threat to crops, resulting in reduced productivity or complete spoilage, which entails substantial economic losses in the agricultural sector. Traditional chemical pesticides can damage soil fertility, contaminate groundwater and aquifers, and release polluting gases into the atmosphere. Moreover, most chemical pesticides are not selective, affecting not only the target organisms but also beneficial species (1), including natural pest predators and crop pollinating insects. Although chemical control may initially seem effective, pest populations often rebound to even higher levels than before the pesticide application (2), as the elimination of natural predators allows pest reproduction to proceed unchecked.

*Xanthomonas* is a genus of gram-negative bacteria that cause over 400 different plant diseases, severely affecting economically important crops such as rice, wheat, citrus, tomato, pepper, cabbage, cassava, banana, and beans (3). *Xanthomonas* can survive in a variety of environments, including soil, seeds, crop remains, and even through interaction with insects. These forms of survival favor the development of epidemics (4). Specifically, *Xanthomonas campestris* pv. *campestris* poses a significant challenge in agriculture. One of the most well-known diseases associated with *X. campestris* is black rot, which affects cruciferous vegetables such as cabbage, broccoli, and cauliflower (5). After entering plants through wounds or natural openings, the bacterium spreads rapidly, causing wilting, yellowing, and eventually blackening of the vascular tissues (6). This can lead to severe economic losses for farmers, as affected crops become unmarketable and may require extensive control measures.

*Xanthomonas* spp. infections can be controlled by cultivating less susceptible species or varieties, implementing strict sanitation practices, and applying copper-based chemicals. However, these measures can be ineffective, and additionally, copper-based pesticides have a negative impact on both health and the environment (4). Therefore, more effective and sustainable measures are needed to control this pathogen.

*Erwinia amylovo*ra, another important gram-negative phytopathogen, causes fire blight, a disease that affects several species of the *Rosaceae* family, including fruit trees such as pear, apple, quince, and loquat, as well as ornamental and wild plants. The damage induced by fire blight is severe, especially in pear trees, and can sometimes lead to the rapid death of the infected plant. The problem is exacerbated by the swift and widespread transmission of the disease and the lack of viable chemical treatments. The most effective approach seems to be the prophylactic application of antibiotics (e.g., streptomycin or oxytetracycline) during the bloom period (7). However, prolonged use of antibiotics is limited by regulatory restrictions, the development of resistance, and concerns about the potential impact on public health when these substances are introduced into the environment (8, 9).

Biological control measures represent a promising alternative approach to minimize or even replace the use of chemicals against plant pathogens, resulting in more sustainable and environmentally friendly agriculture. In this context, bacterial viruses (bacteriophages or phages) have recently been proposed as biocontrol tools against bacteria. Phages infect bacterial cells, propagate inside them, and cause bacterial lysis and death. Potential fields of application include the elimination of bacterial pathogens in human and veterinary medicine, the control of foodborne pathogens in the food industry, and the control of phytopathogens in crop production (10–14).

The innovative nature of phages as biocontrol agents lies in their host specificity, which ensures that other organisms beneficial to soil and plants are not affected. Moreover, they are entirely harmless to plants, humans, and other animals. Another significant advantage is the self-replication capability of phages. As long as sufficient numbers of the target phytopathogen are accessible to the viruses in the soil, the viruses will multiply, infecting and killing their host. Once the phytopathogen disappears or reaches a very low concentration, the phages will cease to spread, become inactive, and ultimately degrade (15).

In this study, bacteriophages against *E. amylovora* and *X. campestris* pv *campestris* were isolated from wastewater samples. These phages were morphologically and genetically characterized, and only virulent phages were selected to assess their efficacy against the phytopathogens *in vitro*. The three phages that yielded the best results in the *in vitro* assays (two against *E. amylovora* and the other against *X. campestris* pv. *campestris*) were tested in infection model experiments using pears for *E. amylovora* and kohlrabi for *X. campestris* pv. *campestris*.

## MATERIALS AND METHODS

### Bacterial strains and culture conditions

The bacterial strains *E. amylovora* NCPPB 595 (CECT 222) and *X. campestris* pv. *campestris* (CECT 97) were used as hosts for the isolation of bacteriophages. Bacteria were grown in nutrient broth (NB; Millipore) at 30°C with shaking. Various bacterial strains were used to test the host range of the isolated phages: *Escherichia coli* strains DH5α (Gibco-BRL, Eggenstein), C600 (ATCC 23724), WG5 (ATCC 700078), and CN13 (ATCC13706), pathogenic *E. coli* O157:H7 strain 933W, environmental isolate *E. coli* ONT, *Shigella sonnei* strain 866 (16), *Salmonella enterica* sv Typhimurium strain WG49 (NCTC 12484), clinical isolates of *S. enterica* sv Enteritidis and *Klebsiella pneumoniae*, *Pseudomonas syringae* pv tomato DC3000, one environmental isolate of *Enterobacter cloacae*, two strains of *Erwinia tasmaniensis*, CECT 17949 and CECT 17950, *Pectobacterium carotovorum (previously Erwinia carotovora*) NCPPB 549 (CECT 314), two non-pathogenic strains of *Xanthomonas arboricora* (CECT 7651 and CECT 7634), and *X. campestris* pv. *citri* (CECT 4428).

### Phage isolation from wastewater samples

For phage isolation, 40 urban wastewater samples were collected from the influent of two urban wastewater treatment plants (WWTPs) in Catalonia (NE Spain). These WWTPs (Besòs and El Prat de Llobregat) serve a combined population of over 2,000,000 inhabitants. Samples were collected in sterile containers, transported to the laboratory within 2 h of collection, and immediately processed for bacteriophage isolation, as described below.

Ten-milliliter samples were filtered through 0.22 µm pore size, low-protein-binding (PES) membranes (Millipore) to remove bacteria and other particulate material. One-milliliter cultures of various phytopathogens at the mid-exponential growth phase (OD_600_ of 0.3) were mixed with 1 mL of the filtered wastewater samples in 8 mL of NB and incubated at 30°C with shaking for 18 h. For subsequent enrichment cultures, 1 mL of the first culture was filtered and used to infect new cultures of the phytopathogens under the same conditions.

### Phage isolation from plaques of lysis

Phage suspensions obtained from wastewater samples were tested against the two phytopathogens using the spot testFeu clic o toqueu aquí per escriure text, culturing the different host strains in NB soft agar (0.7% agar-agar). A 10 µL drop of each phage suspension was directly applied to the solidified lawn of each host strain and allowed to dry prior to incubation. Negative controls were prepared without the addition of phage. Plates were incubated at 30°C for 18 h.

Phage suspensions showing clear, confluent lysis in the spot test were enumerated by the double-layer agar method (17). Briefly, serial decimal dilutions of the phage suspensions in phage buffer (22 mM KH_2_PO_4_, 50 mM Na_2_HPO_4_, 85 mM NaCl, 1 mM MgSO_4_, and 0.1 mM CaCl_2_) were performed to generate isolated plaques of lysis. The dilution selected for plaque isolation provided a higher number of transparent lysis plaques, sufficiently separated to facilitate isolation. Plaques were excised from the soft agar and resuspended in phage buffer. The suspension was treated with chloroform (10%), vortexed for 10 seconds, and centrifuged at 16,000 *× g* for 5 minutes to eliminate bacterial cells. The phages in the supernatant from each isolated plaque were further propagated in larger volumes of the corresponding phytopathogen culture for subsequent analysis. The most optimal condition for phage propagation was established using the phytopathogen culture at the exponential growth phase (OD_600_ of 0.3) and a phage suspension concentration between 10^4^ and 10^5^ PFU/mL.

### Phage particle purification by CsCl density gradients

Suspensions of each individual phage were further purified by cesium chloride (CsCl) density gradients using Ultra Clear thin wall tubes (Beckmann), 1 mL of 20% (wt/vol) sucrose, and three densities of CsCl (1.3, 1.5, and 1.7 g/mL) (18). Samples were ultracentrifuged at 22,000 *× g* for 2 h at 4°C in a Swinging-Bucket SW-41 Rotor in a Beckman ultracentrifuge.

The visible gray bands corresponding to bacteriophages (18) were collected by puncturing the tube, obtaining a 0.5 mL volume that was dialyzed using dialysis membranes (MWC 12–14 kDa, Thermofisher) in dialysis buffer (Tris 0.1 M, EDTA 0.2 mM, pH 8) for 2 h. The dialysis buffer was then replaced with fresh buffer, and dialysis continued for 18 h with magnetic stirring.

### Transmission electron microscopy observation

Ten microliter of concentrated CsCl-purified phage suspensions was dropped onto copper grids with carbon-coated formvar films and negatively stained with 2% ammonium molybdate (pH 7) for 1.5 min. Phages were visualized using a Jeol 1010 transmission electron microscope (TEM; JEOL Inc. Peabody) operating at 80 kV.

### Infectivity assays

To evaluate the dynamics of infection of the isolated phages, selected phage suspensions were used, both individually and in combination, to infect *E. amylovora* or *X. campestris* pv. *campestris* cultures at the mid-exponential growth phase (OD_600_ of 0.3). The infections were carried out at a multiplicity of infection (MOI) of 0.1 and 1 in NB. The culture tubes were incubated at 30°C with shaking, and strain growth or lysis was monitored by measuring the OD_600_. In parallel, the number of colony-forming units per milliliter (CFU/mL) was determined on NB agar, and the number of plaque-forming units per milliliter was obtained by the double agar layer method at 30 min intervals. As a control, a phytopathogen culture without phage was grown under the same conditions. All results represent the average of three to five independent experiments with three replicates for each experiment.

### Infectivity assays in crop models of pears and kohlrabi

Pears (*Pyrus communis* cv. Ercolini, also named Coscia) were disinfected by immersion in 1.5% sodium hypochlorite for 30 min after washing them with sterile distilled water three times. Prior to infection, each pear was wounded using a sterile scalpel, and 10^7^ PFU of ɸEF1 and/or ɸEF2 was applied to the wound. Once this was dry, *E. amylovora* (10^7^ CFU) was added to the wound to infect the fruit. Each pear was placed in a sterile plastic box, which was kept in a controlled environment at 25°C ± 1°C and high relative humidity (60%) for 9 days. NB medium and phage without bacteria were used as negative controls, while bacteria without phage served as positive control.

Plants of kohlrabi (*Brassica oleracea* var. *gongylodes*) were grown in pots in a greenhouse for 8 weeks until each plant had four to five leaves. Leaves were washed with sterile distilled water three times and allowed to dry. Prior to infection, four leaves of each plant were wounded using a needle, and 10^7^ PFU of ɸXF1 (0.1 mL) were sprayed into each wound. Once it was dry, *X. campestris* pv *campestris* (10^7^ CFU) was sprayed onto the wound to infect the plant. The kohlrabi plants were placed in a chamber with a controlled environment at 25°C ± 1°C and high relative humidity (60%) for 20 days. ɸXF1 (10^7^ PFU) was sprayed again onto the wound at days 3 and 5. NB medium and phage without bacteria were used as negative controls, while bacteria without phage served as positive control.

Results were recorded when necrosis was observed in the bacterial control. Necrosis evaluation was performed by image analysis with ImageJ software (19). All assays were conducted on three pears or three kohlrabi leaves per experiment, with each experiment repeated three times independently. For constructing the histograms, the average values of the three replicates from the three independent experiments were used.

### Phage DNA isolation

DNA was isolated from 10^11^ PFU of each phage using the QIAamp DNA blood minikit (Qiagen GmbH), following the manufacturer’s instructions. The DNA was suspended in a final volume of 200 µL of elution buffer. DNA concentration of each sample was evaluated using a Qubit fluorometer with the highly sensitive fluorochrome (2–100 ng) kit. The fluorometer (Life Technologies) results and DNA quality were further confirmed using the 2100 Bioanalyzer system (Agilent Technologies).

### Sequencing, genome reconstruction, and characterization of bacteriophages

Five microliters of DNA at a concentration of 0.2 ng/µL was fragmented and used to prepare libraries for whole-genome sequencing with the Kapa Hyper Plus Kit (Roche), following the manufacturer’s protocol. Libraries were purified using AmPure beads (Beckman Coulter Inc.), checked for fragment distribution and size, and quantified using a TapeStation 4200 and the Agilent High Sensitivity D1000 ScreenTape system (Agilent Technologies) with a Quantus Fluorometer (Promega, WI, US). Phage genomes were separately sequenced on the NextSeq System (Illumina) with a high output run of 300 cycles.

Raw reads were trimmed by Trimmomatic (NexteraPE-PE.fa:2:30:10 LEADING:3 TRAILING:3 SLIDINGWINDOW:4:15 MINLEN:36) (20). The quality of trimmed reads was checked with FastQC (21). Paired-end reads were joined using fq2fa from the idba package (22). Additionally, paired-end filtered reads were assembled individually by SPAdes v3.13.0 (-k 21,33,55,77,99,127) (23). Genome completeness was evaluated using checkcomplete (24) and VIBRANT (25). Open reading frame (ORF) prediction was carried out using Prodigal (26), and functional annotation of predicted genes was performed using Diamond Blastp (27) against the NR NCBI database (February 2022 update) and PHABOX (28). Viral lifestyle was assessed with PhaTYP (29), and the prophage genes in genomes were additionally inspected using Phaster (30) and VIRsorter (31). Genomic maps were generated using Phastest (32). Taxonomic assignment of phage genomes and genome alignments were performed by VIPtree (genome-wide similarity-based) (33). The average nucleotide identity (ANI) of related phage genomes was determined using JSpecies WS.

### Statistical analyses

Statistical analyses were conducted using GraphPad Prism 10 (GraphPad Software). The CFU per milliliter in cultures with phages at each tested time point was log-transformed, and the reduction in the number of cells 3 h post-infection was calculated by comparing to the phage-free cultures. The Shapiro-Wilk and D’Agostino and Pearson normality tests were used to assess data normality. Unpaired *t*-tests were performed to determine differences between infectivity assays. For *in vivo* assays, the pixel values of the necrosis area were obtained using ImageJ. The differences in pixel values of the necrosis area between infected crop models and infected crop models treated with phages were compared using the Mann-Whitney test. A significance cut-off of *P* ≤ 0.05 was used.

## RESULTS AND DISCUSSION

### Isolation of bacteriophages with anti-phytopathogen activity

The phage suspensions obtained from wastewater that showed clear and confluent lysis against the two phytopathogens in the spot test were enumerated by the double-layer agar method. Completely transparent plaques of lysis were selected to obtain pure isolated phage suspensions, as plaque transparency potentially indicates the presence of virulent phages undergoing the lytic cycle. Other selection criteria to guarantee the isolation of different phages were plaque size and morphology. A total of 26 phages were isolated from the wastewater samples: 14 against *E. amylovora* and 12 against *X. campestris* pv. *campestris*.

To obtain enough volume for further analysis and estimate the lytic capacity of the isolated phages, they were propagated in a liquid medium using their respective phytopathogen hosts. A phage was deemed virulent if the culture became transparent after a 5–8 h incubation period (starting OD_600nm_ 0.3). Only phages producing the clearest lysis of the liquid cultures were selected for further analysis, while those that resulted in turbid cultures after infection were discarded. Based on this criterion, the number of isolated phages was reduced to nine against *E. amylovora* and six against *X. campestris* pv. *Campestris*.

### Characterization of the isolated phages

Fourteen isolated phages were purified using CsCl gradients. One phage suspension failed to reach the required concentration (up to 10^7^ PFUs) for purification by this method and was discarded as a possible temperate bacteriophage. The 14 isolated phages were sequenced, but three contained mixed sequences from other phages, preventing reliable assembly despite several purification attempts and were consequently excluded. The sequences of the remaining 11 phages were analyzed, resulting in the selection of two virulent phages against *E. amylovora* (ɸEF1 and ɸEF2) and one virulent phage against *X. campestris* pv. *campestris* (ɸXF1) as candidates for further analysis. As the other phages contained lysogeny-related genes, they were unsuitable for application as antimicrobial tools, given that temperate phages can integrate into host genomes without causing lysis and contribute to horizontal gene transfer (34, 35).

Forty-nine percent of the ORFs in ɸEF2 were found to have a known function, compared to 33.6% in ɸEF1 and 12% in ɸXF1. None of the three phages appeared to be temperate, as no genes encoding lysogeny proteins (e.g., integrase and excisionase) were found among the known ORFs. This aligns with their observed dynamics of infectivity (shown below) and the formation of clear plaques of lysis.

#### Phages ɸEF1 and ɸEF2 infecting Erwinia

**Fig 1:**
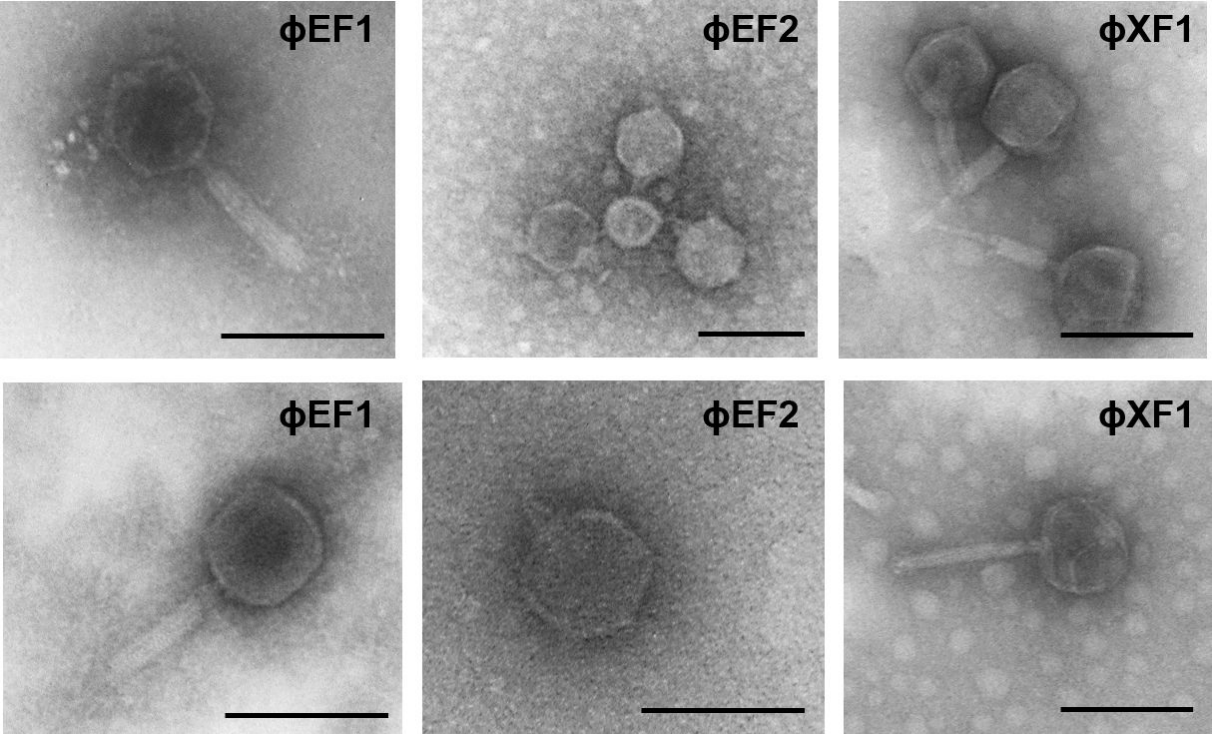
TEMs of phages ɸEF1, ɸEF2, and ɸXF1. Bar, 100 nm.

The two bacteriophages infecting *E. amylovora* were morphologically characterized by TEM. ɸEF1 showed a myovirus-like morphology with head diameters of 77.6 ± 8.9 nm and contractile tails of 114.9 ± 10.2 nm, while ɸEF2 displayed a podovirus-like morphology with head diameters of 74.5 ± 11 nm and short tails of 25.6 ± 0.3 nm (Fig. 1). Both morphologies have been previously described for phages infecting this phytopathogen (36, 37).

**Fig 2:**
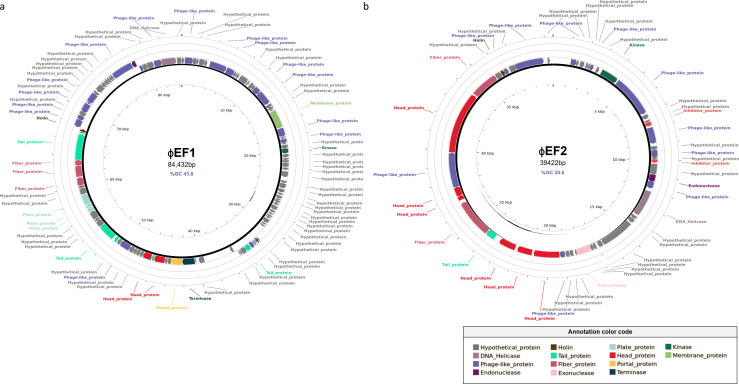
Circular genomic maps of phages ɸEF1 (**a**) and ɸEF2 (**b**) generated with PHASTEST. Each arrow corresponds to an ORF and shows the direction of transcription. ORFs with the same direction of transcription are placed on the same track. Colored arrows represent predicted genes, with each color indicating a specific predicted function. Gray arrows correspond to unidentified ORFs. The legend shows the color code for identified ORFs. Genome size and GC content are indicated within the circle.

A single complete phage genome was assembled from each phage suspension. Phage ɸEF1 (GenBank accession number PP341298) has a dsDNA circular genome of 84,432 bp and 43.8% GC content, with 116 predicted ORFs (Fig. 2a; Dataset S1). Phage ɸEF2 (GenBank accession number PP356685) has a dsDNA genome of 39,422 bp and 50.6% GC content, with 49 predicted ORFs (Fig. 2b; Dataset S1).

**Fig 3:**
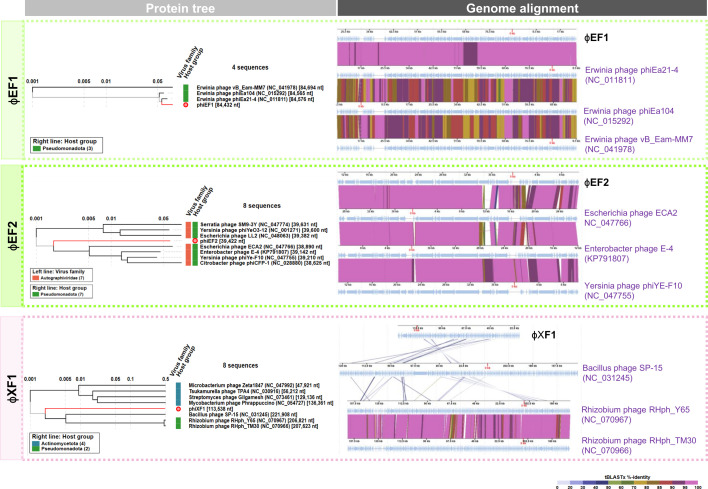
Protein tree and genome alignment of ɸEF1, ɸEF2, and ɸXF1 genomes with the closest relatives in the databases. The viral proteomic tree constructed with ViPTree shows the phylogenetic relationship of each phage with its closest relatives. In the genome alignment, light blue arrows indicate the ORFs. The color bands between the seven genetic maps represent the percentage of identity between each sequence as indicated in the legend at the bottom of the figure.

Genetically, ɸEF1 is most closely related to other *Erwinia* phages (Fig. 3), with *Erwinia* phage phiEa21-4 (NC_011811) showing the highest ANI of 98% over 98.85% of its genome. These results indicate the presence of different genotypes within the same species, as the main species demarcation criterion for bacterial and archaeal viruses, according to the International Committee for the Taxonomy of Viruses, is currently set at a genome sequence identity of 95% over 85% of the complete genome (38).

ɸEF2, on the other hand, is closely related to phages infecting *Enterobacteriaceae* such as *E. coli* (*Escherichia* phage NC_047766) with an ANI of 81% over 83.01% of its genome. ViPTree phylogenetic analysis also places ɸEF2 near phages of *Enterobacter* and *Yersinia* (Fig. 3), but far from other *Erwinia* phages (Fig. S1; Dataset S2).

Regarding the host range, besides *E. amylovora*, phage ɸEF1 was also able to infect *E. tasmaniensis*. Phage ɸEF2 can also infect *E. tasmaniensis,* and as it is related to phages infecting *E. coli* and other *Enterobacteriaceae*, we explored its ability to infect different enterobacteria. ɸEF2 can be described as a broad host range phage, a term applied to phages able to infect multiple species and genera (39), in this case, *E. coli* strains C600, CN13, WG5, and DH5α, and *S. sonnei* strain 866, possibly because they are rough strains that facilitate phage adsorption (40). However, ɸEF2 could not infect pathogenic strains of *E. coli* (serotype O157:H7), environmental isolates of *E. coli* (serotype ONT), clinical isolates of *Salmonella enterica* serovar Typhimurium, *S. enterica* serovar Enteritidis, *Klebsiella pneumoniae*, and environmental isolates of *Pseudomonas syringae* and *Enterobacter cloacae* from our laboratory collection.

Previous reports have documented promiscuous podophages capable of infecting different Gammaproteobacteria (41). Given that *Erwinia* and *E. coli* belong to this bacterial class, albeit to different families (*Erwiniaceae* and *Enterobacteriaceae*, respectively) (42), it is not surprising that ɸEF2 can infect other species within the same class. The ability of phages to infect multiple hosts is considered an evolutionary advantage that ensures the propagation of phage progeny, particularly in environments where the preferred host might not be available. Specifically, broad host range phages of *E. amylovora* have been reported (43), although the phages were only tested against the genera *Pantoea* and *Erwinia* (both in the family *Erwiniaceae*). To the best of our knowledge, this is the first report of a phage capable of infecting such a broad range of Enterobacterales, including *Erwinia (Erwiniaceae* family), *E. coli*, and *S. sonnei* (both in the *Enterobacteriaceae* family). Therefore, the potential applications of ɸEF2 could extend beyond fire blight biocontrol to other enteropathogens, warranting exploration in future studies. Concerning the question of possible phage interference with plant microbiota, bacteria from the *Enterobacteriaceae* family do not appear to be very abundant in fruit trees, being found only in low proportions in the rhizosphere and episphere of the fruit in apple trees (44).

#### Phage ɸXF1 infecting Xanthomonas

**Fig 4:**
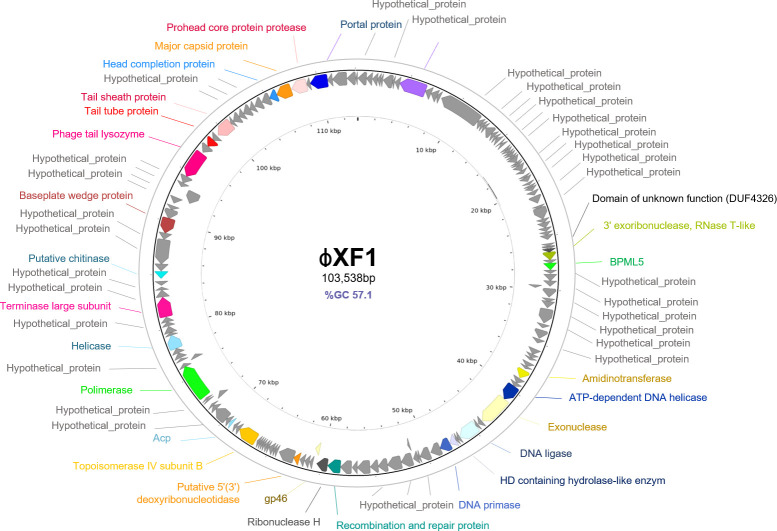
Circular genomic map of phage ɸXF1 generated with VIBRANT. Each arrow corresponds to an ORF and shows the direction of transcription. Colored arrows represent predicted genes, with each color indicating a specific predicted function. Gray arrows correspond to unidentified ORFs. The legend shows the color code for identified ORFs. Genome size and GC content are indicated within the circle.

ɸXF1 exhibits a myovirus-like morphology with head diameters of 75.2 ± 3.5 nm and contractile tails of 128.8 ± 6 nm (Fig. 1). A single complete phage genome was assembled from the phage ɸXF1 suspension. ɸXF1 (GenBank accession number PP475461) has a circular dsDNA genome of 113,538 bp and 57.1% GC content, with 165 predicted ORFs, many of which are identified as hypothetical proteins (Fig. 4; Dataset S1). Although other myovirus-like phages infecting *X. campestris* pv *campestris* have been described (45, 46), ɸXF1 does not resemble any of its closest relatives at the genomic level. The most similar is *Bacillus* phage SP-15 (NC_031245) but sharing an ANI lower than 40%. Phylogenetic analysis by VipTree places ɸXF1 far from previously described *Xanthomonas* phages (Fig. S1).

### *In vitro* antibacterial efficacy of selected phages

**Fig 5:**
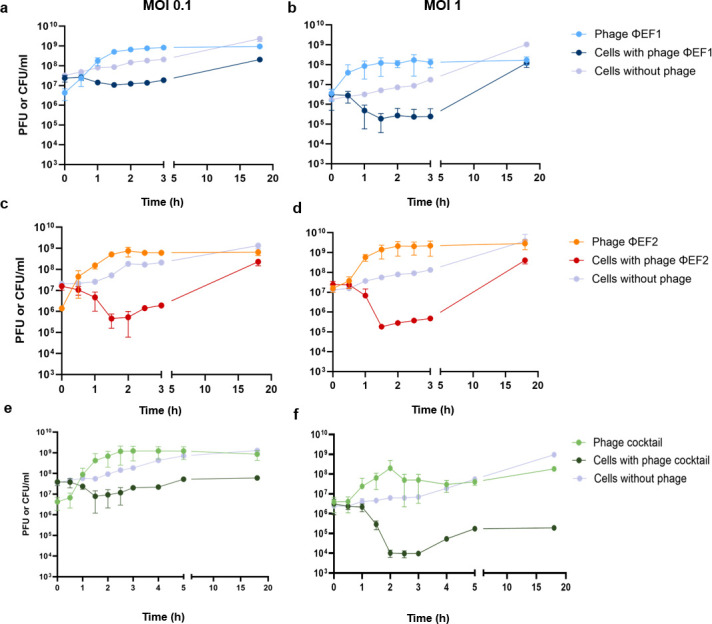
Infectivity dynamics of phages ɸEF1 and ɸEF2. Phage counts (PFU/mL) and *E. amylovora* cell counts (CFU/mL) were monitored over time using each phage individually or in combination (phage cocktail) at MOIs of 0.1 (a,c, and e) and 1 (b, d, and f). Control cells correspond to *E. amylovora* culture without the phages.

**Fig 6:**
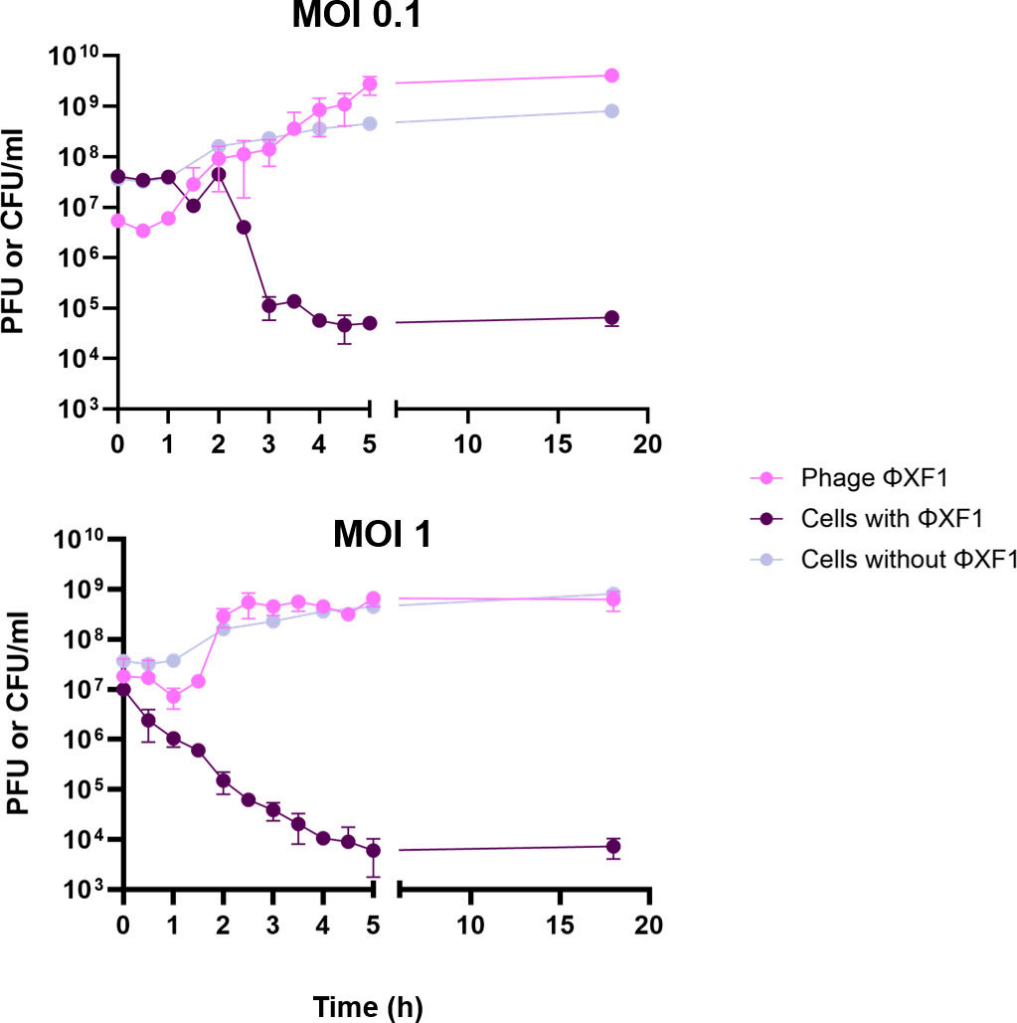
Infectivity dynamics of phage ɸXF1. Phage counts (PFU/mL) and *X. campestris* pv *campestris* cell counts (CFU/mL) were monitored over time at MOIs of 0.1 and 1. Control cells correspond to *X. campestris* pv *campestris* culture without the phage.

Phages ɸEF1 and ɸEF2, assayed individually or in combination (Fig. 5), were used to infect *E. amylovora* cultures at densities close to 10^7^ CFU/mL. Phage ɸXF1 was evaluated alone against an *X. campestris* culture of a similar density (Fig. 6). The capacity of *Erwinia* and *Xanthomonas* phages to infect and reduce their respective host cell numbers was assessed using different MOIs. Here, we describe infections at MOIs of 0.1 and 1, as both were effective in reducing bacterial cell numbers. MOI of 10 was also initially tested and yielded results similar to those of MOI of 1. The infectivity dynamics and bacterial cell number reductions varied between the phages.

Phage ɸEF1 induced a modest reduction of the host strain at an MOI of 0.1, showing a 1.03 log-unit decrease compared to the phage-free control at 3 h and a 0.1 log reduction in cell counts in the presence of the phage from time 0 to 3 h (Fig. 5a). At an MOI of 1, the reduction was more pronounced, with a 1.1 log reduction in cell counts in the presence of the phage after 3 h and a 1.8 log difference compared to the control (Fig. 5b).

Cultures containing phage ɸEF2 achieved a maximal reduction of 2.4 log units compared to the phage-free control after 2 h of infection at an MOI of 0.1 and 1.5 log reduction from time 0 (Fig. 5c). At an MOI of 1, the maximal reduction was 2.6 log units after 3 h, with a 1.7 log-unit reduction in the cell counts in the presence of the phage from time 0 (Fig. 5d).

When both phages were tested in combination (1:1 ratio), only a 1 log-unit reduction compared to the phage-free control was observed in *E. amylovora* cultures containing phages at an MOI of 0.1, with no reduction in the cell counts from time 0 (Fig. 5e). However, when the phage cocktail was used at an MOI of 1, a 3.2 log-unit reduction in the cell counts compared to the phage-free control was observed after 3 h, and the cell counts decreased by 2.5 log units from time 0 (Fig. 5f).

At an MOI of 1, the phage cocktail showed a significantly higher reduction in cell numbers at 3 h compared to the individual phages (ɸEF1 vs cocktail: unpaired *t*-test, *t* = 2.875, *P* value = 0.0452; ɸEF2 vs cocktail: unpaired *t*-test, *t* = 2.873, *P* value = 0.045). However, an increase in the number of cells was observed in all experiments after 3 h, attributed to the emergence of resistant clones. Previous studies have reported a similar regrowth of host strains when using individual phages (47–49), and this tendency can be mitigated by the use of phage cocktails (37, 47). To explore this strategy, we extended the experiment to 20 h at both MOIs. Cell numbers increased by about 1 log unit between 3 and 5 h but stabilized thereafter, with no apparent increase due to the development of resistance. Thus, the phage cocktail not only enhanced the phage bactericidal effect against *E. amylovora* but also curbed the appearance of resistant cells.

Regarding the infectivity dynamics of ɸXF1, after 5 h of infection, this phage achieved a maximal reduction of 3.9 log units in cell counts in the presence of the phage at an MOI of 0.1 and a 4.9 log-units reduction at an MOI of 1 compared to the phage-free control. At the end of the experiment, cell counts in the presence of the phage had decreased by 2.9 log units at an MOI of 0.1 and 3.2 log units at an MOI of 1 (Fig. 6). Cell numbers remained stable from 6 to 20 h after infection at all MOIs tested (data not shown).

Previous studies on isolated phages infecting *E. amylovora* and *X. campestris* have mostly used MOIs of 0.1 and 1 (45, 50–53), although other MOIs have also been explored, ranging from 10 to 100 (37) and even up to 1,000 (48, 51). Consistent with our experiments, MOIs lower than 1 generally did not provide substantial inhibition of the host strain in those studies. For field applications, the use of the lowest possible MOI is advantageous, as high MOIs require the production of large volumes of highly concentrated phage suspensions, which would be impractical and unrealistic. For example, assuming that during a productive infection in a plant, the phytopathogen grows up to 10^8^ cells (54), treating the infection with an MOI of 100–1,000 (as tested in the studies mentioned above) would require a phage suspension containing 10^10−11^ phages/mL.

### Biocontrol assay with the selected phages in vegetable models

After the *in vitro* analyses, the efficacy of phages in preventively inhibiting *E. amylovora* and *X. campestris* pv *campestris* in plants or fruits was assessed. These assays are essential for evaluating the potential application of the selected phages, as many phages that show promising results in reducing viable cell counts of the host bacteria *in vitro* exhibit different properties *in vivo* (36). These differences could be attributed to the variation in phage adsorption kinetics according to the matrix, which may reduce the efficiency of host cell infection. In addition, the efficacy of phage infection may depend on the metabolic state of the host bacterium, which in plants is affected by plant physiology, resistance to the phytopathogen, availability of substrates for the phytopathogen, and environmental conditions (49, 55). These factors can lead to altered gene expression and changes in phage receptors on the bacterial host cell surface, drastically impacting phage infection. For example, *E. amylovora* phage S6 relies on extracellular bacterial cellulose production, as cellulose is its primary receptor for adsorption (49). Environmental factors affecting cellulose biosynthesis, such as oxygen and NO, or during biofilm formation (56) may prevent phage S6 infection.

To date, the interaction of phages with *E. amylovora* has mostly been studied in standard growth media and buffers (47, 57–59). Only a few recent studies report phages that can inhibit *Erwinia* growth in plants using high MOIs (10, 100, and 1,000) with variable efficacy (37, 48, 50). In general, field data demonstrating the biocontrol of fire blight by phages are still preliminary (60).

For *X. campestris*, several infective phages have been isolated and characterized (45, 52, 53, 61), but few studies have evaluated their efficacy in plants (46, 62).

**Fig 7:**
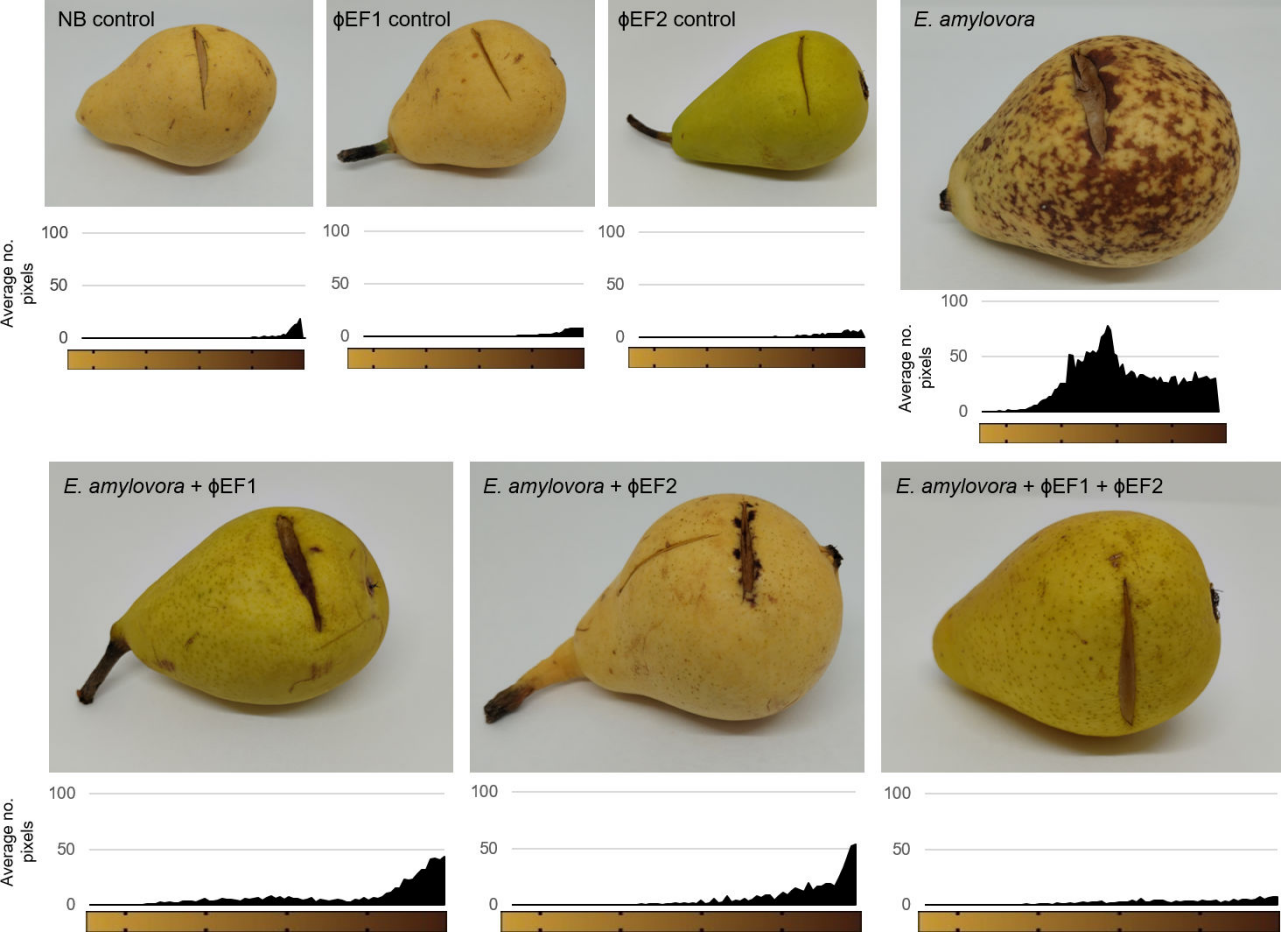
Preventive application of phages ɸEF1 and ɸEF2 against fire blight in a pear model. Pears (*Pyrus communis* cv. Ercolini) were wounded with a sterile scalpel and treated with NB (control), 10^7^ PFU of each phage (ɸEF1 and ɸEF2), 10^7^ CFU of *E. amylovora* (bacterial control), or 10^7^ PFU of the phage cocktail. After allowing the phages to dry, pears were infected with 10^7^ CFU of *E. amylovora*. Pictures were taken after 9 days of incubation and show the result of one representative experiment. Necrosis was monitored using ImageJ software and is represented in the charts below each picture. The histograms were constructed with the average data in the area of infection of three replicates and three independent experiments.

To assess the effect of phages ɸEF1 and ɸEF2 on *E. amylovora* disease, we used pears as a model. Three sets of individual pears were wounded, infected with 10^7^ CFU of *E. amylovora*, and treated with phages at an MOI of 1 in three independent experiments. The best incubation conditions for the experiments were found to be 25°C ± 1°C and high relative humidity (60%). These conditions allowed us to attribute the color changes to the bacterial infection rather than to the oxidation of the pear after being cut. After 9 days, the pears infected with *E. amylovora* (bacterial control) showed clear fire blight symptoms (Fig. 7). When phages ɸEF1 and ɸEF2 were used preventively (applied before bacterial infection), necrosis was drastically reduced. Histogram analysis of images revealed that fire blight lesions (Fig. 7) were smaller in phage-treated pears (Table S1), and no significant differences were observed between the treatments with one phage or the other (Table S3). However, the reduction in necrosis was even higher when both phages were applied together (Fig. 7; Table S1). Although individual phage treatments appeared to work effectively, the phage cocktail was associated with a reduced occurrence of spontaneous bacterial resistance during the infectivity assays, indicating this is a better strategy for fire blight biocontrol.

**Fig 8:**
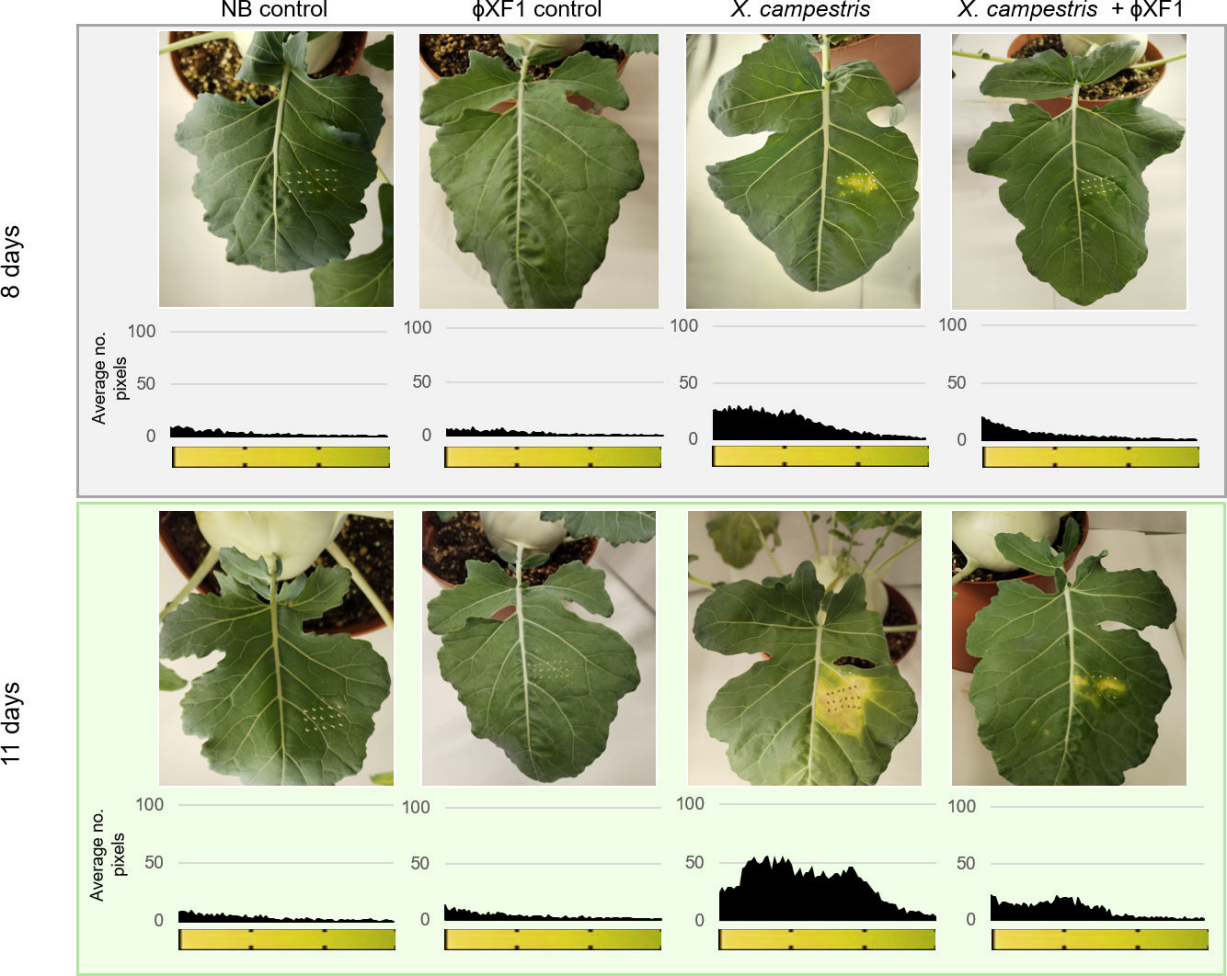
Preventive application of phage ɸXF1 against black rot in a kohlrabi model. Kohlrabi plants (*Brassica oleracea* var. *gongylodes*) were punctured using a sterile needle and treated with NB (control), 10^7^ PFU of phage ɸXF1, or 10^7^ CFU of *X. campestris* pv *campestris* (bacterial control). After allowing the phage to dry, kohlrabi plants were infected with 10^7^ CFU of *X. campestris* pv *campestris*. Pictures were taken after 8 and 11 days of incubation and show the result of one representative experiment. Necrosis was monitored using ImageJ software and is represented in the charts below each picture. The histograms were constructed with the average data in the area of infection of three replicates and three independent experiments.

The efficacy of phage ɸXF1 in controlling black rot was initially tested under laboratory conditions using *ex vivo* leaves from various *Brassica* species. However, this approach proved unsuccessful as the leaves rotted before showing clear signs of the disease, indicating the model was unsuitable for studying this phytopathogen. As *X. campestris* pv *campestris* infects through the colonization of xylem vessels (63), it needs to be tested on living plants with an active vascular system. Given that some *Brassicaceae* can be challenging to handle in controlled incubation conditions, kohlrabi was selected for its manageable size in the laboratory. The incubation period lasted 20 days, but clear black rot symptoms appeared as early as day 8 after infection with the phytopathogen (Fig. 8; Table S2). In the control (bacteria only), necrosis became more pronounced 3 days later (day 11), after which no significant changes in the extent of the necrotic area were observed for the remainder of the experiment (until day 20).

In contrast, treatment with ɸXF1 before infecting the plant clearly reduced disease symptoms in the leaves (Table S2). At day 8 of incubation, no necrosis was observed in any of the replicates, and at day 11, necrosis was markedly reduced compared to the control (Fig. 8; Table S3), this being the day with the most evident signs of disease in the phage-treated leaves. Furthermore, while all control leaves infected with *X. campestris* pv *campestris* had similar levels of necrosis, some of the phage-treated leaf replicates showed no signs of necrosis at day 11 or later. In these cases, a better distribution of phage particles on the leaves was likely responsible for almost or completely inhibiting the development of black rot. From day 11 to the end of the experiment (day 20), no changes in the extent of necrosis, if present, were observed in phage-treated leaves. As with fire blight, image analysis of kohlrabi leaves confirmed the visual observations of disease progression in the absence/presence of ɸXF1 (Fig. 8).

Only a few phage-based products are currently available on the market for plant disease control, such as AgriPhage in the US (https://www.omnilytics.com/agriculture/), Erwiphage PLUS (Enviroinvest) produced in Hungary (36), or Biolyse in Scotland (https://www.apsbiocontrol.com). Despite the demonstrated efficacy of these products in certain areas, it is advisable to develop new alternatives, ideally containing viruses capable of infecting strains present in a range of geographical regions. This would ensure effective disease reduction when applied in different countries. Although broad-spectrum phages can be isolated, many phages have a more restricted host range. In general, the presence of the host strain will determine the abundance of the specific associated phages, and therefore, some products may be more effective than others depending on the region and strain.

To optimize phage-based products and improve their efficacy for disease control, several factors need to be considered. These include gaining a better understanding of phage-pathogen dynamics and determining the optimal application window. Additionally, evaluating and managing the best strategy for applying the product to plant surfaces for protection before pathogen colonization or for curative purposes once disease development has started is crucial. However, in the latter situation, legislation often requires affected trees to be cut down. Optimally, cocktails of several phages should be used, as host cells resistant to one phage may be sensitive to others, as observed here with the combined use of *E. amylovora* phages. Additionally, the impact of phages on plant physiology and microbiota should be monitored. Even though phages do not directly interact with plants, they can impact beneficial strains in the rhizosphere and phyllosphere. Changes in microbial communities can impact plant growth and nutrient uptake (64). While some studies suggest that phage treatment does not affect the plant microbiome (60), others indicate that changes can occur (36).

## Data Availability

The genomic data generated in this study are publicly available in GenBank under accession numbers PP341298, PP356685, and PP475461.

## SUPPLEMENTAL MATERIAL

Dataset S1 spectrum.00280-25-s0001.xlsx Phages annotation.

Dataset S2 spectrum.00280-25-s0002.xlsx ViptTree scores.

Figure S1 spectrum.00280-25-s0003.pdf Viral proteomic tree constructed with ViPTree.

Supplemental tables spectrum.00280-25-s0004.pdf Quantitative data of the *in vivo* experiments.

ASM does not own the copyrights to Supplemental Material that may be linked to, or accessed through, an article. The authors have granted ASM a non-exclusive, world-wide license to publish the Supplemental Material files. Please contact the corresponding author directly for reuse.
