# Supplementary material for: Efficacy of Erwinia amylovora and Xanthomonas campestris pv campestris phages to control fire blight and black rot in vivo
Source: Microbiol Spectr. 2025 May 16;13(7):e00280-25. doi: 10.1128/spectrum.00280-25 (PMC12211020; doi:10.1128/spectrum.00280-25)
Supplement: Figure S1 — Viral proteomic tree constructed with ViPTree. [file spectrum.00280-25-s0003.pdf]

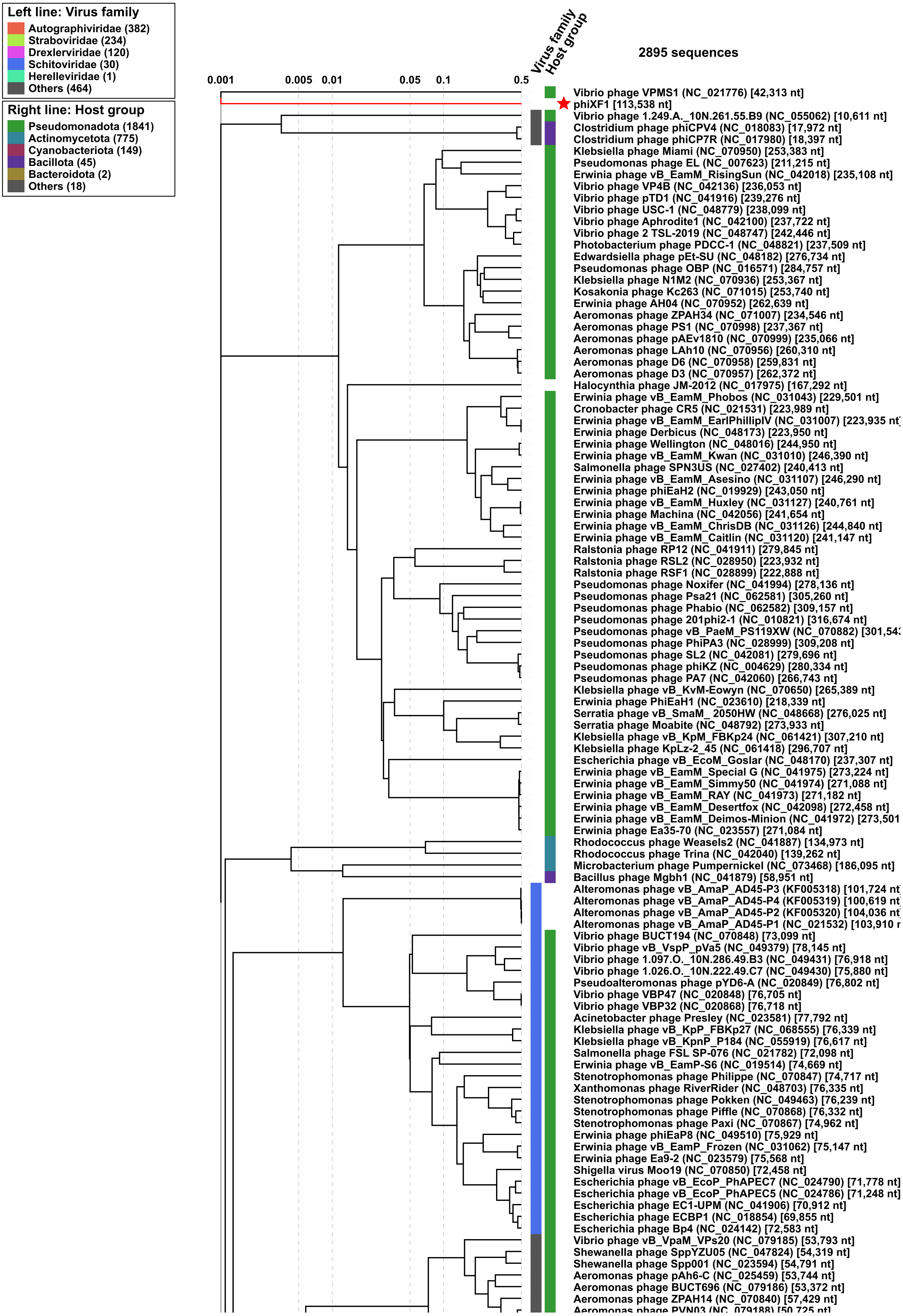
Left line: Virus family
Autographiviridae (382)
Straboviridae (234)
Drexlerviridae (120)
Schitoviridae (30)
Herelleviridae (1)
Others (464)
Right line: Host group
Pseudomonadota (1841)
Actinomycetota (775)
Cyanobacteriota (149)
Bacillota (45)
Bacteroidota (2)
Others (18)
Virus family
Host group
2895 sequences
0.001
0.005
0.01
0.05
0.1
0.5
Vibrio phage VPMS1 (NC_021776) [42,313 nt]
phiXF1 [113,538 nt]
Vibrio phage 1.249.A._10N.261.55.B9 (NC_055062) [10,611 nt]
Clostridium phage phiCPV4 (NC_018083) [17,972 nt]
Clostridium phage phiCP7R (NC_017980) [18,397 nt]
Klebsiella phage Miami (NC_070950) [253,383 nt]
Pseudomonas phage EL (NC_007623) [211,215 nt]
Erwinia phage vB_EamM_RisingSun (NC_042018) [235,108 nt]
Vibrio phage VP4B (NC_042136) [236,053 nt]
Vibrio phage pTD1 (NC_041916) [239,276 nt]
Vibrio phage USC-1 (NC_048779) [238,099 nt]
Vibrio phage Aphrodite1 (NC_042100) [237,722 nt]
Vibrio phage 2 TSL-2019 (NC_048747) [242,446 nt]
Photobacterium phage PDCC-1 (NC_048821) [237,509 nt]
Edwardsiella phage pEt-SU (NC_048182) [276,734 nt]
Pseudomonas phage OBP (NC_016571) [284,757 nt]
Klebsiella phage N1M2 (NC_070936) [253,367 nt]
Kosakonia phage Kc263 (NC_071015) [253,740 nt]
Erwinia phage AH04 (NC_070952) [262,639 nt]
Aeromonas phage ZPAH34 (NC_071007) [234,546 nt]
Aeromonas phage PS1 (NC_070998) [237,367 nt]
Aeromonas phage pAEv1810 (NC_070999) [235,066 nt]
Aeromonas phage LAh10 (NC_070956) [260,310 nt]
Aeromonas phage D6 (NC_070958) [259,831 nt]
Aeromonas phage D3 (NC_070957) [262,372 nt]
Halocynthia phage JM-2012 (NC_017975) [167,292 nt]
Erwinia phage vB_EamM_Phobos (NC_031043) [229,501 nt]
Cronobacter phage CR5 (NC_021531) [223,989 nt]
Erwinia phage vB_EamM_EarlPhillipIV (NC_031007) [223,935 nt]
Erwinia phage Derbicus (NC_048173) [223,950 nt]
Erwinia phage Wellington (NC_048016) [244,950 nt]
Erwinia phage vB_EamM_Kwan (NC_031010) [246,390 nt]
Salmonella phage SPN3US (NC_027402) [240,413 nt]
Erwinia phage vB_EamM_Asesino (NC_031107) [246,290 nt]
Erwinia phage phiEaH2 (NC_019929) [243,050 nt]
Erwinia phage vB_EamM_Huxley (NC_031127) [240,761 nt]
Erwinia phage Machina (NC_042056) [241,654 nt]
Erwinia phage vB_EamM_ChrisDB (NC_031126) [244,840 nt]
Erwinia phage vB_EamM_Caitlin (NC_031120) [241,147 nt]
Ralstonia phage RP12 (NC_041911) [279,845 nt]
Ralstonia phage RSL2 (NC_028950) [223,932 nt]
Ralstonia phage RSF1 (NC_028899) [222,888 nt]
Pseudomonas phage Noxifer (NC_041994) [278,136 nt]
Pseudomonas phage Psa21 (NC_062581) [305,260 nt]
Pseudomonas phage Phabio (NC_062582) [309,157 nt]
Pseudomonas phage 201phi2-1 (NC_010821) [316,674 nt]
Pseudomonas phage vB_PaeM_PS119XW (NC_070882) [301,54
Pseudomonas phage PhiPA3 (NC_028999) [309,208 nt]
Pseudomonas phage SL2 (NC_042081) [279,696 nt]
Pseudomonas phage phiKZ (NC_004629) [280,334 nt]
Pseudomonas phage PA7 (NC_042060) [266,743 nt]
Klebsiella phage vB_KvM-Eowyn (NC_070650) [265,389 nt]
Erwinia phage PhiEaH1 (NC_023610) [218,339 nt]
Serratia phage vB_SmaM_ 2050HW (NC_048668) [276,025 nt]
Serratia phage Moabite (NC_048792) [273,933 nt]
Klebsiella phage vB_KpM_FBKp24 (NC_061421) [307,210 nt]
Klebsiella phage KpLz-2_45 (NC_061418) [296,707 nt]
Escherichia phage vB_EcoM_Goslar (NC_048170) [237,307 nt]
Erwinia phage vB_EamM_Special G (NC_041975) [273,224 nt]
Erwinia phage vB_EamM_Simmy50 (NC_041974) [271,088 nt]
Erwinia phage vB_EamM_RAY (NC_041973) [271,182 nt]
Erwinia phage vB_EamM_Desertfox (NC_042098) [272,458 nt]
Erwinia phage vB_EamM_Deimos-Minion (NC_041972) [273,501
Erwinia phage Ea35-70 (NC_023557) [271,084 nt]
Rhodococcus phage Weasels2 (NC_041887) [134,973 nt]
Rhodococcus phage Trina (NC_042040) [139,262 nt]
Microbacterium phage Pumpernickel (NC_073468) [186,095 nt]
Bacillus phage Mgbh1 (NC_041879) [58,951 nt]
Alteromonas phage vB_AmaP_AD45-P3 (KF005318) [101,724 nt
Alteromonas phage vB_AmaP_AD45-P4 (KF005319) [100,619 nt
Alteromonas phage vB_AmaP_AD45-P2 (KF005320) [104,036 nt
Alteromonas phage vB_AmaP_AD45-P1 (NC_021532) [103,910 r
Vibrio phage BUCT194 (NC_070848) [73,099 nt]
Vibrio phage vB_VspP_pVa5 (NC_049379) [78,145 nt]
Vibrio phage 1.097.O._10N.286.49.B3 (NC_049431) [76,918 nt]
Vibrio phage 1.026.O._10N.222.49.C7 (NC_049430) [75,880 nt]
Pseudoalteromonas phage pYD6-A (NC_020849) [76,802 nt]
Vibrio phage VBP47 (NC_020848) [76,705 nt]
Vibrio phage VBP32 (NC_020868) [76,718 nt]
Acinetobacter phage Presley (NC_023581) [77,792 nt]
Klebsiella phage vB_KpP_FBKp27 (NC_068555) [76,339 nt]
Klebsiella phage vB_KpnP_P184 (NC_055919) [76,617 nt]
Salmonella phage FSL SP-076 (NC_021782) [72,098 nt]
Erwinia phage vB_EamP-S6 (NC_019514) [74,669 nt]
Stenotrophomonas phage Philippe (NC_070847) [74,717 nt]
Xanthomonas phage RiverRider (NC_048703) [76,335 nt]
Stenotrophomonas phage Pokken (NC_049463) [76,239 nt]
Stenotrophomonas phage Piffle (NC_070868) [76,332 nt]
Stenotrophomonas phage Paxi (NC_070867) [74,962 nt]
Erwinia phage phiEaP8 (NC_049510) [75,929 nt]
Erwinia phage vB_EamP_Frozen (NC_031062) [75,147 nt]
Erwinia phage Ea9-2 (NC_023579) [75,568 nt]
Shigella virus Moo19 (NC_070850) [72,458 nt]
Escherichia phage vB_EcoP_PhAPEC7 (NC_024790) [71,778 nt]
Escherichia phage vB_EcoP_PhAPEC5 (NC_024786) [71,248 nt]
Escherichia phage EC1-UPM (NC_041906) [70,912 nt]
Escherichia phage ECBP1 (NC_018854) [69,855 nt]
Escherichia phage Bp4 (NC_024142) [72,583 nt]
Vibrio phage vB_VpaM_VPs20 (NC_079185) [53,793 nt]
Shewanella phage SppYZU05 (NC_047824) [54,319 nt]
Shewanella phage Spp001 (NC_023594) [54,791 nt]
Aeromonas phage pAh6-C (NC_025459) [53,744 nt]
Aeromonas phage BUCT696 (NC_079186) [53,372 nt]
Aeromonas phage ZPAH14 (NC_070840) [57,429 nt]
Aeromonas phage PVN03 (NC_079188) [50,725 nt]

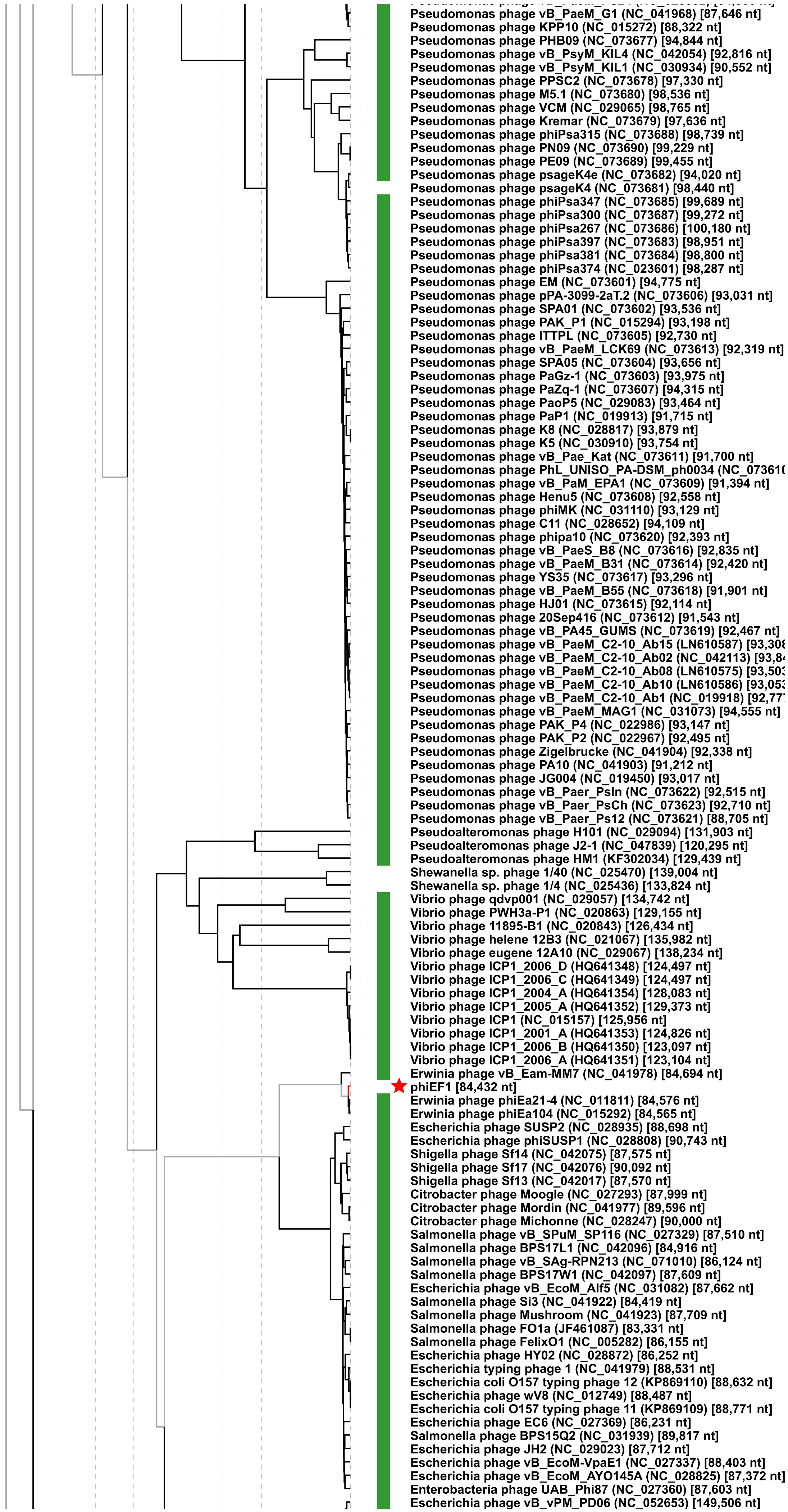
Pseudomonas phage vB_PaeM_G1 (NC_041968) [87,646 nt]
Pseudomonas phage KPP10 (NC_015272) [88,322 nt]
Pseudomonas phage PHB09 (NC_073677) [94,844 nt]
Pseudomonas phage vB_PsyM_KIL4 (NC_042054) [92,816 nt]
Pseudomonas phage vB_PsyM_KIL1 (NC_030934) [90,552 nt]
Pseudomonas phage PPSC2 (NC_073678) [97,330 nt]
Pseudomonas phage M5.1 (NC_073680) [98,536 nt]
Pseudomonas phage VCM (NC_029065) [98,765 nt]
Pseudomonas phage Kremar (NC_073679) [97,636 nt]
Pseudomonas phage phiPsa315 (NC_073688) [98,739 nt]
Pseudomonas phage PN09 (NC_073690) [99,229 nt]
Pseudomonas phage PE09 (NC_073689) [99,455 nt]
Pseudomonas phage psageK4e (NC_073682) [94,020 nt]
Pseudomonas phage psageK4 (NC_073681) [98,440 nt]
Pseudomonas phage phiPsa347 (NC_073685) [99,689 nt]
Pseudomonas phage phiPsa300 (NC_073687) [99,272 nt]
Pseudomonas phage phiPsa267 (NC_073686) [100,180 nt]
Pseudomonas phage phiPsa397 (NC_073683) [98,951 nt]
Pseudomonas phage phiPsa381 (NC_073684) [98,800 nt]
Pseudomonas phage phiPsa374 (NC_023601) [98,287 nt]
Pseudomonas phage EM (NC_073601) [94,775 nt]
Pseudomonas phage pPA-3099-2aT.2 (NC_073606) [93,031 nt]
Pseudomonas phage SPA01 (NC_073602) [93,536 nt]
Pseudomonas phage PAK_P1 (NC_015294) [93,198 nt]
Pseudomonas phage ITTPL (NC_073605) [92,730 nt]
Pseudomonas phage vB_PaeM_LCK69 (NC_073613) [92,319 nt]
Pseudomonas phage SPA05 (NC_073604) [93,656 nt]
Pseudomonas phage PaGz-1 (NC_073603) [93,975 nt]
Pseudomonas phage PaZq-1 (NC_073607) [94,315 nt]
Pseudomonas phage PaoP5 (NC_029083) [93,464 nt]
Pseudomonas phage PaP1 (NC_019913) [91,715 nt]
Pseudomonas phage K8 (NC_028817) [93,879 nt]
Pseudomonas phage K5 (NC_030910) [93,754 nt]
Pseudomonas phage vB_Pae_Kat (NC_073611) [91,700 nt]
Pseudomonas phage PhL_UNISO_PA-DSM_ph0034 (NC_07361
Pseudomonas phage vB_PaM_EPA1 (NC_073609) [91,394 nt]
Pseudomonas phage Henu5 (NC_073608) [92,558 nt]
Pseudomonas phage phiMK (NC_031110) [93,129 nt]
Pseudomonas phage C11 (NC_028652) [94,109 nt]
Pseudomonas phage phipa10 (NC_073620) [92,393 nt]
Pseudomonas phage vB_PaeS_B8 (NC_073616) [92,835 nt]
Pseudomonas phage vB_PaeM_B31 (NC_073614) [92,420 nt]
Pseudomonas phage YS35 (NC_073617) [93,296 nt]
Pseudomonas phage vB_PaeM_B55 (NC_073618) [91,901 nt]
Pseudomonas phage HJ01 (NC_073615) [92,114 nt]
Pseudomonas phage 20Sep416 (NC_073612) [91,543 nt]
Pseudomonas phage vB_PA45_GUMS (NC_073619) [92,467 nt]
Pseudomonas phage vB_PaeM_C2-10_Ab15 (LN610587) [93,30
Pseudomonas phage vB_PaeM_C2-10_Ab02 (NC_042113) [93,8
Pseudomonas phage vB_PaeM_C2-10_Ab08 (LN610575) [93,50
Pseudomonas phage vB_PaeM_C2-10_Ab10 (LN610586) [93,05
Pseudomonas phage vB_PaeM_C2-10_Ab1 (NC_019918) [92,77
Pseudomonas phage vB_PaeM_MAG1 (NC_031073) [94,555 nt]
Pseudomonas phage PAK_P4 (NC_022986) [93,147 nt]
Pseudomonas phage PAK_P2 (NC_022967) [92,495 nt]
Pseudomonas phage Zigelbrucke (NC_041904) [92,338 nt]
Pseudomonas phage PA10 (NC_041903) [91,212 nt]
Pseudomonas phage JG004 (NC_019450) [93,017 nt]
Pseudomonas phage vB_Paer_PsIn (NC_073622) [92,515 nt]
Pseudomonas phage vB_Paer_PsCh (NC_073623) [92,710 nt]
Pseudomonas phage vB_Paer_Ps12 (NC_073621) [88,705 nt]
Pseudoalteromonas phage H101 (NC_029094) [131,903 nt]
Pseudoalteromonas phage J2-1 (NC_047839) [120,295 nt]
Pseudoalteromonas phage HM1 (KF302034) [129,439 nt]
Shewanella sp. phage 1/40 (NC_025470) [139,004 nt]
Shewanella sp. phage 1/4 (NC_025436) [133,824 nt]
Vibrio phage qdvp001 (NC_029057) [134,742 nt]
Vibrio phage PWH3a-P1 (NC_020863) [129,155 nt]
Vibrio phage 11895-B1 (NC_020843) [126,434 nt]
Vibrio phage helene 12B3 (NC_021067) [135,982 nt]
Vibrio phage eugene 12A10 (NC_029067) [138,234 nt]
Vibrio phage ICP1_2006_D (HQ641348) [124,497 nt]
Vibrio phage ICP1_2006_C (HQ641349) [124,497 nt]
Vibrio phage ICP1_2004_A (HQ641354) [128,083 nt]
Vibrio phage ICP1_2005_A (HQ641352) [129,373 nt]
Vibrio phage ICP1 (NC_015157) [125,956 nt]
Vibrio phage ICP1_2001_A (HQ641353) [124,826 nt]
Vibrio phage ICP1_2006_B (HQ641350) [123,097 nt]
Vibrio phage ICP1_2006_A (HQ641351) [123,104 nt]
Erwinia phage vB_Eam-MM7 (NC_041978) [84,694 nt]
phiEF1 [84,432 nt]
Erwinia phage phiEa21-4 (NC_011811) [84,576 nt]
Erwinia phage phiEa104 (NC_015292) [84,565 nt]
Escherichia phage SUSP2 (NC_028935) [88,698 nt]
Escherichia phage phiSUSP1 (NC_028808) [90,743 nt]
Shigella phage Sf14 (NC_042075) [87,575 nt]
Shigella phage Sf17 (NC_042076) [90,092 nt]
Shigella phage Sf13 (NC_042017) [87,570 nt]
Citrobacter phage Moogle (NC_027293) [87,999 nt]
Citrobacter phage Mordin (NC_041977) [89,596 nt]
Citrobacter phage Michonne (NC_028247) [90,000 nt]
Salmonella phage vB_SPuM_SP116 (NC_027329) [87,510 nt]
Salmonella phage BPS17L1 (NC_042096) [84,916 nt]
Salmonella phage vB_SAg-RPN213 (NC_071010) [86,124 nt]
Salmonella phage BPS17W1 (NC_042097) [87,609 nt]
Escherichia phage vB_EcoM_Alf5 (NC_031082) [87,662 nt]
Salmonella phage Si3 (NC_041922) [84,419 nt]
Salmonella phage Mushroom (NC_041923) [87,709 nt]
Salmonella phage FO1a (JF461087) [83,331 nt]
Salmonella phage FelixO1 (NC_005282) [86,155 nt]
Escherichia phage HY02 (NC_028872) [86,252 nt]
Escherichia typing phage 1 (NC_041979) [88,531 nt]
Escherichia coli O157 typing phage 12 (KP869110) [88,632 nt]
Escherichia phage wV8 (NC_012749) [88,487 nt]
Escherichia coli O157 typing phage 11 (KP869109) [88,771 nt]
Escherichia phage EC6 (NC_027369) [86,231 nt]
Salmonella phage BPS15Q2 (NC_031939) [89,817 nt]
Escherichia phage JH2 (NC_029023) [87,712 nt]
Escherichia phage vB_EcoM-VpaE1 (NC_027337) [88,403 nt]
Escherichia phage vB_EcoM_AYO145A (NC_028825) [87,372 nt]
Enterobacteria phage UAB_Phi87 (NC_027360) [87,603 nt]
Escherichia phage vB_vPM_PD06 (NC_052653) [149,506 nt]

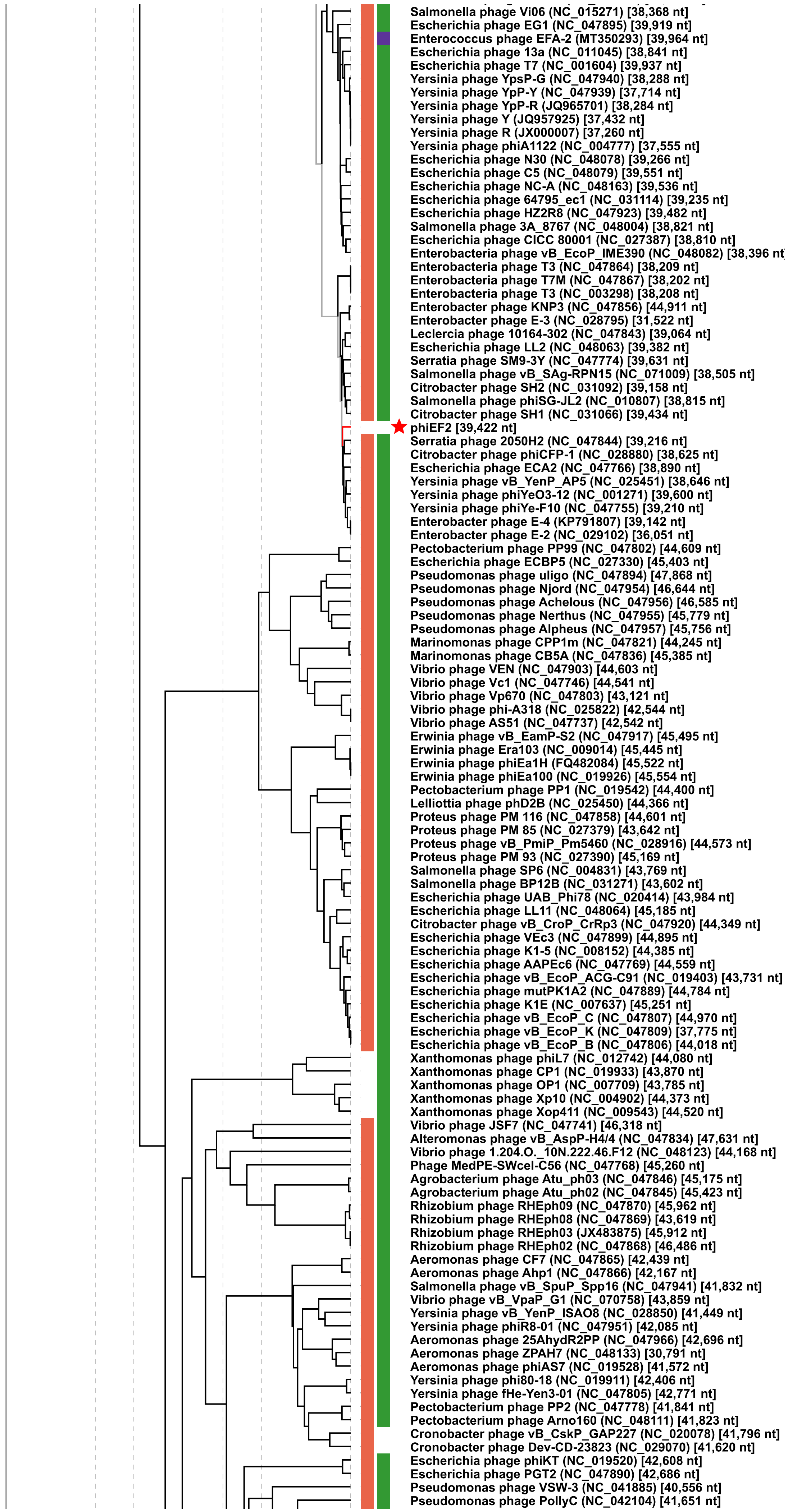
Salmonella phage Vi06 (NC_015271) [38,368 nt]
Escherichia phage EG1 (NC_047895) [39,919 nt]
Enterococcus phage EFA-2 (MT350293) [39,964 nt]
Escherichia phage 13a (NC_011045) [38,841 nt]
Escherichia phage T7 (NC_001604) [39,937 nt]
Yersinia phage YpsP-G (NC_047940) [38,288 nt]
Yersinia phage YpP-Y (NC_047939) [37,714 nt]
Yersinia phage YpP-R (JQ965701) [38,284 nt]
Yersinia phage Y (JQ957925) [37,432 nt]
Yersinia phage R (JX000007) [37,260 nt]
Yersinia phage phiA1122 (NC_004777) [37,555 nt]
Escherichia phage N30 (NC_048078) [39,266 nt]
Escherichia phage C5 (NC_048079) [39,551 nt]
Escherichia phage NC-A (NC_048163) [39,536 nt]
Escherichia phage 64795_ec1 (NC_031114) [39,235 nt]
Escherichia phage HZ2R8 (NC_047923) [39,482 nt]
Salmonella phage 3A_8767 (NC_048004) [38,821 nt]
Escherichia phage CICC 80001 (NC_027387) [38,810 nt]
Enterobacteria phage vB_EcoP_IME390 (NC_048082) [38,396 nt
Enterobacteria phage T3 (NC_047864) [38,209 nt]
Enterobacteria phage T7M (NC_047867) [38,202 nt]
Enterobacteria phage T3 (NC_003298) [38,208 nt]
Enterobacter phage KNP3 (NC_047856) [44,911 nt]
Enterobacter phage E-3 (NC_028795) [31,522 nt]
Leclercia phage 10164-302 (NC_047843) [39,064 nt]
Escherichia phage LL2 (NC_048063) [39,382 nt]
Serratia phage SM9-3Y (NC_047774) [39,631 nt]
Salmonella phage vB_SAg-RPN15 (NC_071009) [38,505 nt]
Citrobacter phage SH2 (NC_031092) [39,158 nt]
Salmonella phage phiSG-JL2 (NC_010807) [38,815 nt]
Citrobacter phage SH1 (NC_031066) [39,434 nt]
phiEF2 [39,422 nt]
Serratia phage 2050H2 (NC_047844) [39,216 nt]
Citrobacter phage phiCFP-1 (NC_028880) [38,625 nt]
Escherichia phage ECA2 (NC_047766) [38,890 nt]
Yersinia phage vB_YenP_AP5 (NC_025451) [38,646 nt]
Yersinia phage phiYeO3-12 (NC_001271) [39,600 nt]
Yersinia phage phiYe-F10 (NC_047755) [39,210 nt]
Enterobacter phage E-4 (KP791807) [39,142 nt]
Enterobacter phage E-2 (NC_029102) [36,051 nt]
Pectobacterium phage PP99 (NC_047802) [44,609 nt]
Escherichia phage ECBP5 (NC_027330) [45,403 nt]
Pseudomonas phage uligo (NC_047894) [47,868 nt]
Pseudomonas phage Njord (NC_047954) [46,644 nt]
Pseudomonas phage Achelous (NC_047956) [46,585 nt]
Pseudomonas phage Nerthus (NC_047955) [45,779 nt]
Pseudomonas phage Alpheus (NC_047957) [45,756 nt]
Marinomonas phage CPP1m (NC_047821) [44,245 nt]
Marinomonas phage CB5A (NC_047836) [45,385 nt]
Vibrio phage VEN (NC_047903) [44,603 nt]
Vibrio phage Vc1 (NC_047746) [44,541 nt]
Vibrio phage Vp670 (NC_047803) [43,121 nt]
Vibrio phage phi-A318 (NC_025822) [42,544 nt]
Vibrio phage AS51 (NC_047737) [42,542 nt]
Erwinia phage vB_EamP-S2 (NC_047917) [45,495 nt]
Erwinia phage Era103 (NC_009014) [45,445 nt]
Erwinia phage phiEa1H (FQ482084) [45,522 nt]
Erwinia phage phiEa100 (NC_019926) [45,554 nt]
Pectobacterium phage PP1 (NC_019542) [44,400 nt]
Lelliottia phage phD2B (NC_025450) [44,366 nt]
Proteus phage PM 116 (NC_047858) [44,601 nt]
Proteus phage PM 85 (NC_027379) [43,642 nt]
Proteus phage vB_PmiP_Pm5460 (NC_028916) [44,573 nt]
Proteus phage PM 93 (NC_027390) [45,169 nt]
Salmonella phage SP6 (NC_004831) [43,769 nt]
Salmonella phage BP12B (NC_031271) [43,602 nt]
Escherichia phage UAB_Phi78 (NC_020414) [43,984 nt]
Escherichia phage LL11 (NC_048064) [45,185 nt]
Citrobacter phage vB_CroP_CrRp3 (NC_047920) [44,349 nt]
Escherichia phage VEc3 (NC_047899) [44,895 nt]
Escherichia phage K1-5 (NC_008152) [44,385 nt]
Escherichia phage AAPEc6 (NC_047769) [44,559 nt]
Escherichia phage vB_EcoP_ACG-C91 (NC_019403) [43,731 nt]
Escherichia phage mutPK1A2 (NC_047889) [44,784 nt]
Escherichia phage K1E (NC_007637) [45,251 nt]
Escherichia phage vB_EcoP_C (NC_047807) [44,970 nt]
Escherichia phage vB_EcoP_K (NC_047809) [37,775 nt]
Escherichia phage vB_EcoP_B (NC_047806) [44,018 nt]
Xanthomonas phage phiL7 (NC_012742) [44,080 nt]
Xanthomonas phage CP1 (NC_019933) [43,870 nt]
Xanthomonas phage OP1 (NC_007709) [43,785 nt]
Xanthomonas phage Xp10 (NC_004902) [44,373 nt]
Xanthomonas phage Xop411 (NC_009543) [44,520 nt]
Vibrio phage JSF7 (NC_047741) [46,318 nt]
Alteromonas phage vB_AspP-H4/4 (NC_047834) [47,631 nt]
Vibrio phage 1.204.O._10N.222.46.F12 (NC_048123) [44,168 nt]
Phage MedPE-SWcel-C56 (NC_047768) [45,260 nt]
Agrobacterium phage Atu_ph03 (NC_047846) [45,175 nt]
Agrobacterium phage Atu_ph02 (NC_047845) [45,423 nt]
Rhizobium phage RHEph09 (NC_047870) [45,962 nt]
Rhizobium phage RHEph08 (NC_047869) [43,619 nt]
Rhizobium phage RHEph03 (JX483875) [45,912 nt]
Rhizobium phage RHEph02 (NC_047868) [46,486 nt]
Aeromonas phage CF7 (NC_047865) [42,439 nt]
Aeromonas phage Ahp1 (NC_047866) [42,167 nt]
Salmonella phage vB_SpuP_Spp16 (NC_047941) [41,832 nt]
Vibrio phage vB_VpaP_G1 (NC_070758) [43,859 nt]
Yersinia phage vB_YenP_ISAO8 (NC_028850) [41,449 nt]
Yersinia phage phiR8-01 (NC_047951) [42,085 nt]
Aeromonas phage 25AhydR2PP (NC_047966) [42,696 nt]
Aeromonas phage ZPAH7 (NC_048133) [30,791 nt]
Aeromonas phage phiAS7 (NC_019528) [41,572 nt]
Yersinia phage phi80-18 (NC_019911) [42,406 nt]
Yersinia phage fHe-Yen3-01 (NC_047805) [42,771 nt]
Pectobacterium phage PP2 (NC_047778) [41,841 nt]
Pectobacterium phage Arno160 (NC_048111) [41,823 nt]
Cronobacter phage vB_CskP_GAP227 (NC_020078) [41,796 nt]
Cronobacter phage Dev-CD-23823 (NC_029070) [41,620 nt]
Escherichia phage phiKT (NC_019520) [42,608 nt]
Escherichia phage PGT2 (NC_047890) [42,686 nt]
Pseudomonas phage VSW-3 (NC_041885) [40,556 nt]
Pseudomonas phage PollyC (NC_042104) [41,651 nt]

Supplementary material legends

Supplementary Figure 1. Protein tree of ϕEF1, ϕEF2 and ϕXF1 genomes with whole viral database. Viral proteomic tree constructed with ViPTree shows the phylogenetic relationship of each phage with its closest relatives.
