# Supplementary material for: Efficacy of Erwinia amylovora and Xanthomonas campestris pv campestris phages to control fire blight and black rot in vivo
Source: Microbiol Spectr. 2025 May 16;13(7):e00280-25. doi: 10.1128/spectrum.00280-25 (PMC12211020; doi:10.1128/spectrum.00280-25)
Supplement: Supplemental tables — Quantitative data of the in vivo experiments. [file spectrum.00280-25-s0004.pdf]

Supplementary tables

Table S1. Total number of pixels (brown range, dark) in the necrosis area of the infected pears.

Sample	Average	SD
E. amylovora + ϕEF-1	527.20	318.73
E. amylovora + ϕEF-2	354.00	235.25
E. amylovora + ϕEF-1 + ϕEF-2	77.23	14.34
E. amylovora	1776.25	476.24
Control ϕEF-1	40.90	28.00
Control ϕEF-2	53.67	57.55
Control NB	18.00	14.00

Table S2. Total number of pixels (yellow range, light) in the necrosis area of the infected kohlrabies.

Sample	Average	SD
X. campestris + ϕXF-1 (8 days)	393.00	322.30
X. campestris (8 days)	1266.00	574.28
Control ϕXF-1 (8 days)	221.00	126.89
Control NB (8 days)	191.00	76.92
X. campestris + ϕXF-1 (11 days)	469.50	375.50
X. campestris (11 days)	1759.00	365.93
Control ϕXF-1 (11 days)	262.67	187.95
Control NB (11 days)	288.67	88.55

Table S3. Statistical differences between infected crop models and infected crop models treated with phages.

	Mann-Whitney U	P value	Significantly different
ϕEF1 vs *E. amylovora*	0	0.0286	Yes
ϕEF2 vs *E. amylovora*	0	0.0286	Yes
ϕEF1 vs ϕEF2	2	0.1143	No
Cocktail vs *E. amylovora*	0	0.0286	Yes
Cocktail vs ϕEF1	2	0.0286	Yes
Cocktail vs ϕEF2	0	0.0286	Yes
ϕXF1 vs *X. campestris* (day 8)	3	0.0177	Yes
ϕXF1 vs *X. campestris* (day 11)	0	0.0061	Yes

The differences in pixel values of the necrosis area between infected crop models and infected crop models treated with phages were compared using the Mann-Whitney test.
